# Acute and chronic allograft dysfunction following pediatric lung transplantation

**DOI:** 10.1016/j.jhlto.2026.100498

**Published:** 2026-01-23

**Authors:** Afsoon Sepahzad, Matthew Thomas, Malcolm Brodlie, Paul Aurora, Helen Spencer, Rossa Brugha

**Affiliations:** a Paediatric Respiratory Medicine, Great Ormond Street Hospital, London, UK; b Paediatric Respiratory Medicine, Great North Children's Hospital, Newcastle upon Tyne Hospitals NHS Foundation Trust, Newcastle upon Tyne, UK; c Translational and Clinical Research Institute, Faculty of Medical Sciences, Newcastle University, Newcastle upon Tyne, UK; d Respiratory Medicine and Cardiothoracic Transplantation, Great Ormond Street Hospital, London, UK; e Infection, Immunity and Inflammation, UCL, Great Ormond Street Institute of Child Health, London, UK

**Keywords:** pediatric lung transplant, acute allograft dysfunction, chronic allograft dysfunction, rejection, transplantation

## Abstract

**Background:**

Pediatric lung transplantation is a life-saving procedure for children with end-stage lung disease. As immunosuppression and treatment have improved, so too have survival outcomes; however, pediatric lung transplant recipients still face challenges, including acute rejection, infection, and long-term issues such as chronic lung allograft dysfunction (CLAD).

**Methods:**

This review details early complications following pediatric lung transplantation and provides an explorative investigation of the mechanisms underlying acute allograft dysfunction, and current prevention and treatment strategies. Focus is then given to CLAD with an overview of the subtypes of CLAD, review of recognized risk factors, challenges around diagnostics, current treatment strategies, and importantly future research goals to aid in the better identification and characterization of CLAD.

**Conclusions:**

As our understanding of acute and CLAD has evolved over time so have prevention and treatment strategies, including the assessment and treatment of risk factors such as infection and aspiration. The diagnosis of CLAD, however, remains extremely challenging with limited treatment options. Future research should focus on the prevention of CLAD.

## Background

Pediatric lung transplantation is a life-saving procedure for children with end-stage lung disease. Lung transplantation offers the potential for improved quality of life and extended survival. The estimated median survival for pediatric lung transplant recipients from international data is 6 years, with a median conditional survival of 9.1 years, compared to 8.3 years in adults.^1^ As understanding of post lung transplant care has evolved, improved postoperative follow-up and immunosuppression strategies, and a multidisciplinary approach have contributed to improved survival rates. However, pediatric lung transplant recipients still face challenges, including acute rejection, infection, and long-term issues such as chronic lung allograft dysfunction (CLAD). The most recent International Society for Heart & Lung Transplantation (ISHLT) report highlights that graft failure is the most common cause of death in the first 30 days.^2^ After 30 days, and up to 1 year post transplant, infections are the primary cause of death, followed by graft failure. The incidence of acute lung allograft rejection is reported as 30% to 50%^3^ during the first-year post transplant, accounting for 3.8% of deaths in the first month. Beyond 1 year, CLAD accounts for approximately 40% of deaths in pediatric post-transplant patients.^4^ CLAD remains the main obstacle to long-term survival after lung transplant.^5^ Due to limited studies in the pediatric population, some references in this review are taken from adult transplant cohorts. Where reference is made to pediatric studies, this has been highlighted.

## Early complications

### Primary graft dysfunction

Primary graft dysfunction (PGD) is the primary cause of early mortality after lung transplantation. PGD (also referred to as early graft dysfunction or severe ischemia-reperfusion injury) reflects acute lung injury and is similar in phenotype to pediatric acute respiratory distress syndrome. PGD typically occurs in the first 72 hours post lung transplantation. An acute inflammatory cascade results in respiratory epithelial cell damage, and subsequent chronic inflammation may contribute to the development of CLAD.^6^

Chest radiography and PaO_2_/FiO_2_ ratio are used to grade the severity of the syndrome^7^ with grade 3 PGD the most severe form, defined by the presence of alveolar infiltrates and a ratio of PaO_2_ (mm Hg):FiO_2_ less than 200. Table 1 summarizes the diagnostic criteria which were revised by the ISHLT in 2016.^7^

**Table 1:** Diagnostic Criteria for Primary Graft Dysfunction as Outlined in the Revised ISHLT Consensus Group Statement 2016

Grade	Presence of pulmonary edema on chest X-ray	PaO_2_/FiO_2_ ratio
0	No	Any
1	Yes	>300
2	Yes	200-300
3	Yes	<200

Abbreviations: ISHLT, the International Society for Heart & Lung Transplantation.

The causes of PGD are multifactorial and include donor-related, recipient-related, and perioperative risk factors. In adult patients, risk factors such as donor tobacco use, obese recipients, or recipient history of pulmonary hypertension and single lung transplant have been shown to increase the risk of PGD.^8^ There are currently no standardized therapies for preventing PGD. Treatment is aimed at maintenance of negative fluid balance, the use of pulmonary vasodilator therapy, and lung-protective ventilation.^9^ In the pediatric population, there is evidence to suggest that earlier use of extracorporeal membrane oxygenation postoperatively may result in improved outcomes by avoiding higher ventilator pressures and possible barotrauma.^10^ The impact of cold-ischemic time, the interval between donor cross clamp (cardiac arrest for donation after circulatory death cases), and the longest reperfusion time in the recipient on PGD remains unclear. Earlier studies suggested cold-ischemic time as a possible risk factor for PGD.^11^ Later studies, however, found no effect of ischemic time on the development of PGD.^12^, ^13^

### Reflux-aspiration

Following pediatric lung transplantation, complications include gastro-esophageal reflux due to impairment of cough reflexes and mucociliary clearance,^14^ esophageal dysmotility secondary to vagal nerve injury, ischemia and local scarring,^15^ and impaired gastric motility^16^ thought to be secondary to vagal nerve injury, or possibly due to the effects of immunosuppressive drugs.^17^ Gastro-esophageal reflux is associated with increased risk of developing CLAD.^18^ The use of calcineurin inhibitors has been shown to exacerbate reflux,^19^ and postoperative gastroparesis may promote microaspiration into the lung allograft.^16^ Patients without evidence of reflux have been shown to have improved survival, and fundoplication appears to reduce the risk of long-term allograft dysfunction.^20^, ^21^ This highlights the importance of objective assessments of reflux utilizing multichannel intraluminal impedance in addition to pH testing, ideally during the pretransplant assessment, or early post-transplant.^22^ The first prospective study of reflux/aspiration immediately post transplantation looked at the Reflux Symptom Index score and results of manometry and pH/impedance. Testing was performed on a maintenance proton pump inhibitor. Bronchoalveolar lavage (BAL) fluid was assessed for pepsin, bile salts, IL-8, and neutrophils. The detection of pepsin suggested aspiration and proximal reflux events correlated with neutrophilia, linked to allograft dysfunction and mortality.^23^ There is also evidence to suggest that early fundoplication (<6 months post transplantation) is associated with higher forced expiratory volume in 1 second (FEV1) 5 years after lung transplantation.^24^ The benefits, however, need to be balanced against the risks of further surgery and anesthesia for a patient in the early period following transplantation. In preparing this review, it was not possible to find published data on individual center practice variation, or outcomes, related to investigation or treatment choices in gastro-esophageal reflux, and this appears to be an area where this patient group may be underserved.

A recent review of lung transplant–induced diaphragmatic palsy among adults highlighted important potential impacts, including significantly increased duration of invasive ventilatory support, use of noninvasive ventilation, and increased length of intensive care unit stay, including readmissions, few studies however explored the association with CLAD.^25^ Two single-center studies found that having a diaphragmatic palsy was not a contributing factor to developing CLAD or advanced PGD.^26^, ^27^

### Infection

Infection is a significant risk for patients in the early post-transplant period due to high levels of immune suppression. The highest rates of bloodstream infection occur within the first 30 days following transplantation with over half occurring in the first 7 days, and early bloodstream infections are associated with increased risk of death in the first-year post transplantation in children.^28^ Ensuring vaccinations are up to date is also an important part of pretransplant management.^29^

Gram-negative bacterial pathogens, including *Pseudomonas*, *Klebsiella*, and *Haemophilus* species, may cause pneumonia in the postoperative period.^30^ An antibiotic regimen, pre, during, and immediately post-transplant, should be tailored to the individual patient’s previously identified pathogens; for example, dual antipseudomonal IV antibiotics are usually chosen for pediatric patients with cystic fibrosis.^31^

Cytomegalovirus (CMV) is common and patients at risk of developing the most severe primary infection include those who are CMV negative receiving lungs from CMV-positive donors.^32^ A single episode of CMV pneumonitis is associated with an increased risk of mortality or retransplantation within the first year.^33^ The presence of CMV infection is usually determined by conventional viral culture, pp65 antigenemia testing, shell vial viral culture, or CMV polymerase chain reaction. CMV syndrome comprises a constellation of symptoms including fever, fatigue, thrombocytopenia, and/or leukopenia in the presence of confirmed infection. Invasive disease is proven through histologic proof of CMV inclusions in biopsy tissue in association with clinical signs and symptoms.^33^ Other manifestations of CMV disease include small bowel or gastric disease, renal disease, and retinitis. Prophylaxis with ganciclovir can be used for patients with CMV mismatch. In those not needing prophylaxis with ganciclovir, acyclovir is used as prophylaxis for herpes simplex infections. While the optimal duration of prophylaxis is debated, there is clear evidence to demonstrate that the risk for CMV is increased in the immediate period after stopping prophylaxis, with the greatest risk in the first 6 weeks.^34^ This demonstrates the importance of increased surveillance during this time. In view of potential nephrotoxicity and drug interactions with immunosuppressive agents, a pre-emptive approach may be employed in place of antiviral prophylaxis, which involves close monitoring for CMV viraemia (with regular blood CMV polymerase chain reactions (PCRs)) and prompt initiation of antivirals if CMV is detected.^35^ Newer anti-CMV agents, including maribavir and letermovir, are emerging for treatment of resistant/refractory CMV, as alternatives to foscarnet and cidofovir.^36^

Data from adult lung transplant recipients suggest that community-acquired viruses can also cause significant morbidity and mortality, and adenovirus infection is associated with an increased incidence of early graft failure and death.^37^ While the mainstay of treatment for viral infections is supportive, there is some evidence to support the use of inhaled or intravenous ribavirin in severe cases.^38^ The use of cidofovir for adenovirus, and ribavirin for respiratory syncytial virus and human metapneumovirus in the pediatric population appears to be safe and effective,^39^, ^40^ combination therapies with newer antivirals may also be considered.^41^ The Clinical Trials in Organ Transplant in Children Study (CTOTC-03), a major multicenter study across 6 pediatric institutions in the United States, found that, in contrast to the adult lung transplant recipients, respiratory viral infections in pediatric lung transplant recipients do not appear to be a predictor of poor outcome.^42^

Influenza vaccination is the mainstay of influenza disease prevention and has been shown to reduce morbidity and mortality in transplant recipients.^43^ Neuraminidase inhibitors, including oral oseltamivir, are approved for the treatment of influenza, and early use is associated with reduced risk of mortality in pediatric transplant patients.^44^ Importantly, unlike in adult lung transplant patients, children, and adolescents are at a low risk for severe COVID-19.^45^ Data for the use of antivirals and monoclonal antibodies in the pediatric transplant population are limited.

Fungal infections carry a significant risk of morbidity and mortality. Invasive *Aspergillus* may confer up to 60% mortality in post-transplant patients.^46^ *Candida albicans* is frequently detected and while it may reflect colonization, due to the potential of invasive disease, it is frequently treated, most centers will use nystatin prophylaxis. Treatment of fungal infections depends on the organism identified and sensitivities. Importantly, -azole antifungals demonstrate interaction with tacrolimus and careful drug monitoring is required when starting and stopping these agents.

## Rejection

There are several forms of acute lung rejection seen in lung transplant recipients. Hyperacute rejection appears in the immediate post-transplant period. Subsequently, acute cellular rejection (ACR) and antibody-mediated rejection (AMR) may be seen. ACR is more frequently observed. ACR and AMR can occur within days or even years following lung transplantation.^47^

### Types of acute rejection

Hyperacute rejection is an early and severe complication of transplantation, triggered by the presence of preformed antibodies in the recipient that are directed against the donor. This form of rejection occurs within minutes to hours after transplantation, as the antibodies recognize donor-specific antigens, typically the human leukocyte antigen (HLA) system or other surface markers found on endothelial cells or tissues of the donor lung. Upon binding to the donor tissue, these antibodies activate the complement system, leading to direct cytotoxic lysis.^48^ The primary consequences include damage to the microvasculature and thrombosis, which impairs oxygenation, causing respiratory failure and irreversible graft injury.

ACR is predominantly T-cell mediated and involves recognition of non-self HLA or other antigens.^49^ Two distinct pathways of allorecognition are implicated. Dendritic cells migrate from the graft to secondary lymphoid tissue to present histocompatibility complex directly to recipient T-cells via the direct pathway.^50^ In the indirect pathway, recipient dendritic cells process and present alloantigen from donor antigen-presenting cells to T-cells, either in secondary lymphoid tissue or in the allograft. Following allorecognition, T-cells undergo clonal expansion and differentiate into alloreactive cytotoxic T-cells.^51^ These CD8+ T-cells migrate to the graft, bind to major histocompatibility complex molecules on the allograft, and initiate tissue destruction.^50^ The incidence of ACR is reported as 17% to 55% in the first-year post transplantation.^52^

AMR occurs following complement-mediated lysis or antibody-dependent cellular toxicity. This can arise due to preformed antibodies, or via de novo recipient antibodies which have formed in response to donor antigens following allorecognition by native antigen-presenting cells. AMR is, in part, defined by the measurement of circulating donor-specific antibody (DSA). These are defined as class I (antibodies against HLA-A, -B, -C, found on all nucleated cells) and class II (antibodies against HLA-DP, -DQ, -DR, found on B-cells, monocytes, and dendritic cells).^53^ Class II DSA, particularly those against the DQ locus, carry a higher risk of subsequent CLAD and mortality compared to class I DSA, or non–donor-specific HLA antibodies.^54^, ^55^

The end-point of both of these processes is initiation of a local inflammatory response comprised of direct cell-mediated killing (via induced apoptosis), cell lysis via the complement cascade, and recruitment of effector cells following release of chemokines, including interferons and tissue necrosis factor. This results in an inflamed, edematous, microthrombosed alveolar-capillary unit, with impaired gas exchange, and may progress to fibrosis and permanent damage of the graft.

### Prevention of acute rejection

HLA matching is the primary means by which the risk of immediate rejection is minimized. Perioperative strategies focus on immune modulation to prevent rejection. While protocols may vary between centers, they generally include a calcineurin inhibitor (such as cyclosporine or tacrolimus) to block the synthesis of IL-2 and other cytokines produced by activated T-cells,^56^ corticosteroids, and a cell cycle inhibitor (mycophenolate mofetil) to prevent lymphocyte proliferation.^57^ Registry data highlight a rise in the rates of induction immunosuppression—with almost 75% of recipients receiving induction therapy from January 2010 to June 2018, a significant increase from 64% in 2005-2009. The majority received polyclonal antilymphocyte or antithymocyte globulin, and interleukin-2 receptor antagonists used in 18% of recipients. There was no survival difference seen in pediatric lung transplant recipients receiving or not receiving induction therapy,^58^ and the evidence supporting the use of induction immunosuppression remains limited.^59^ There is evidence to suggest a role for rituximab induction in preventing early DSA development in pediatric lung transplant recipients without adverse effects and may improve outcomes.^60^

### Presentation of acute rejection

Clinical detection of ACR and AMR can be challenging as the presentation can be variable. Some pediatric patients are entirely asymptomatic, and evidence of rejection is only detected on routine surveillance,^61^ while others may present with nonspecific symptoms similar to infection (cough, low-grade fever, malaise), making clinical diagnosis difficult, and chest radiographs may be normal. A decrease in percent predicted forced expiratory volume in 1 second (ppFEV1) is often the main finding, with a 10% decline in ppFEV1 quoted as a threshold to intervene/investigate.^62^

### Investigation for acute rejection

Following transplantation, BAL is carried out for the purpose of surveillance or for diagnostic purposes. The ISHLT BAL survey identified that surveillance bronchoscopies with BAL were typically performed by 2 weeks and at 3-, 6-, and 12-months post lung transplant.^63^ The recommendation for minimum time points for surveillance bronchoscopies with BAL is 1-, 3-, 6-, and 12-month post-transplant, and to consider additional time points in view of the challenges in performing surveillance pulmonary function tests in this population. Diagnostic bronchoscopies are recommended when there is clinical concern for lower airway infection.

Diagnosis of ACR is made by examining transbronchial biopsy specimens, with histological findings of mononuclear cell infiltration of arterioles and bronchioles.^47^ Biopsies are predominantly taken from the lower lobes, as the grade of rejection is reported to be higher than from contemporaneous upper lobe biopsies.^64^ Acute rejection has been detected in up to one third of biopsies taken as part of routine surveillance during the first year.^65^ In 2007, the ISHLT published a revision of the “working formulation for the standardization of nomenclature in the diagnosis of lung rejection” which provided diagnostic criteria for ACR.^66^ ACR is divided into vascular (arteriolar/capillary) rejection “A grade” and small airways rejection/lymphocytic bronchiolitis “B grade.” The grade of acute rejection increases as the cellular infiltrate becomes more extensive. There is intraobserver and interobserver variability in the interpretation of ACR by pathologists.^67^ Infection can result in similar histological features as ACR, therefore exclusion of concurrent infection is crucial,^66^ BAL samples should be sent for extensive microbiological analysis, including culture of atypical organisms, PCR for an extended respiratory viral panel, including CMV and Epstein-Barr virus, PCR for *Pneumocystis jirovecii*. Centers may also send samples for molecular testing and metagenomics.

### Treatment of acute cellular rejection

ACR is a significant risk factor for developing CLAD, with evidence to suggest an increased risk with only a single episode.^5^, ^68^, ^69^, ^70^ First-line treatment is with steroids, usually intravenous methylprednisolone. Treatment of grade A1 rejection may vary between centers and depends on physician's choice.^71^, ^72^ Treatment of recurrent or persistent ACR is challenging. Options include repeated courses of steroids, switching to tacrolimus in place of cyclosporine, and considering other immunosuppressive agents including anti-interleukin-2 receptor antagonists, polyclonal antithymococyte globulin or muromonab-CD3.^47^ Alemtuzumab, an anti-CD52 monoclonal antibody, has also been shown to demonstrate some efficacy in refractory cases.^73^ A small (*n* = 15) study of azithromycin following a diagnosis of lymphocytic bronchiolitis suggests improved inflammatory markers, although there was no control group data for comparison.^74^

### Diagnosis of antibody-mediated rejection

The International Society for Heart and Lung Transplantation has determined diagnostic criteria and a working consensus definition,^75^ with AMR defined on the presence or absence of allograft dysfunction, histology suggestive of AMR (small vessel vasculitis) and positive C4d staining on biopsy, and positive DSA. The likelihood of diagnosis is graded as “possible,” “probable,” and “definite” on the basis of there being 1, 2, or 3 of those factors present. Non-HLA antibodies have also been of interest in recent studies. Notably, antibodies against type V collagen, K-alpha 1 tubulin, and angiotensin type 1 receptor (anti-AT1R), which are expressed on airway epithelial and endothelial cells, have been associated with PGD and development of bronchiolitis obliterans syndrome (BOS) phenotype of CLAD (BOS).^76^ Staining for C4d presents with several challenges with the sensitivity and specificity of C4d positivity in lung biopsies being much less reliable than data published for other solid organ transplants.^77^ The specificity of C4d staining in the lung allograft is also limited by deposition in the context of infection and preservation injury.^78^

### Treatment of antibody-mediated rejection

Due to the challenges in making a diagnosis and limited clinical trials, there is no standardized treatment for AMR.^79^ First-line therapy is targeted to antibody removal via plasmapheresis and suppression of antibody production with intravenous immunoglobulin therapy. Second-line treatments remain experimental, options include rituximab, eculizumab, bortezomib, tocilizumab, and daratumumab.^80^, ^81^, ^82^

## Chronic lung allograft dysfunction

CLAD is an umbrella term encompassing 4 phenotypes that together describe persistent graft dysfunction following transplantation after exclusion of other potential causes.^3^ After the first-year post-transplant, CLAD is the most common cause of death.^83^

Historically, BOS was the ascribed term for chronic failure of the graft following lung transplantation. It has subsequently been recognized that other distinct spirometric and radiological phenotypes of chronic graft failure exist, which led to the description of CLAD as an overarching term. BOS accounts for approximately 70% of CLAD. The other phenotypes currently described are restrictive allograft syndrome (RAS), “mixed,” and “undefined.”^84^

### Definition of CLAD

CLAD is defined as an absolute FEV1 decline of >20% from baseline, where the baseline is the average of the 2 best post-transplant values for FEV1 obtained ≥3 weeks apart. The duration of decline determines whether it is labeled as “possible CLAD” (<3 weeks), “probable CLAD” (FEV1 decline for ≥3 weeks), and “definite CLAD” (FEV1 decline ≥3 months). A separate label of “Potential CLAD” is considered when the FEV1 decline is ≥10% from baseline.

### Diagnosis and investigations for CLAD

**Figure 1:**
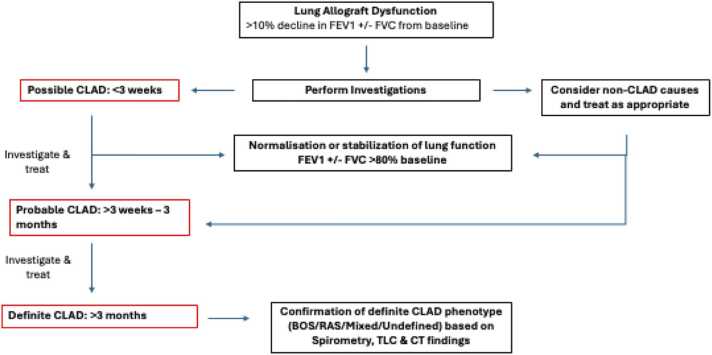
The evolution of CLAD and key time points for investigation. BOS, bronchiolitis obliterans syndrome; CLAD, chronic lung allograft dysfunction; CT, computerized tomography; FEV1, forced expiratory volume in 1 second; FVC, forced vital capacity; RAS, restrictive allograft syndrome; TLC, total lung capacity.

CLAD is a diagnosis of exclusion, and recognition of potential CLAD should trigger investigations of possible causes, including full pulmonary function tests, chest imaging (computed tomography) with inspiratory and expiratory phase imaging, in addition to blood analysis of infection markers and DSAs.^3^ Bronchoscopy, lavage, and biopsies should be performed to investigate for infection, acute rejection, and to look at lavage white cell populations for phenotyping. Gastroesophageal reflux disease and/or aspiration should be excluded as contributory factors. A CLAD diagnosis, defined by absolute FEV1 in liters, makes interpretation and diagnosis difficult for pediatricians. Our patients may not be able to do spirometry, lung and thoracic cage growth mean that a “baseline” absolute FEV1 is a moving target, lobar transplants from adults can grow when transplanted into children,^85^ and differences in reference equations can impact our ability to use ppFEV1 across centers.^86^ Specific airflows can be measured in children in an effort-independent fashion during multiple breath washout, including lung clearance index as well as S_acin_ and S_cond_.^87^ There is evidence to suggest that lung clearance index in the pediatric cohort may be a more sensitive means of detecting lung allograft dysfunction than the use of standard spirometry.^88^ Figure 1, adapted from the ISHLT Consensus report in 2019, demonstrates the evolution of CLAD highlighting time points for investigation.^5^

### Risk factors and pathophysiology of CLAD

Several risk factors have been suggested for the development of CLAD. These are alloantigen-dependent (cellular-mediated rejection and AMR) and alloantigen-independent factors (infection, aspiration, ischemia, and autoimmunity).^84^ The pathophysiology of CLAD is multifactorial. Immune activation is the main driver, provoked by both alloimmune and nonalloimmune triggers.^3^ Development of CLAD is believed to be related to the number and severity of “hits” post-transplant.^3^ There is strong evidence to demonstrate the association between acute rejection episodes and the development of CLAD^5^, ^68^ and the impact of infections and subsequent CLAD.^89^ Community-acquired respiratory viruses have a significant impact, with one report finding a 40% rate of BOS among lung transplant recipients surviving paramyxovirus or adenovirus infection.^90^ Bacterial and fungal infections are also identified as risk factors with *Pseudomonas aeruginosa, Staphylococcus aureus*, and *Aspergillus* increasingly recognized as important risk factors for CLAD.^91^

There is a wealth of evidence to support the use of prophylactic azithromycin to prevent the development of CLAD.^92^, ^93^, ^94^ Treatment with Azithromycin prophylaxis has been shown to reduce long-term CLAD prevalence, improve CLAD-free survival, pulmonary function, and functional exercise capacity following lung transplant.^94^ However, in practice, indications for use of Azithromycin, timing, and dosing vary across lung transplant centers.^95^

### CLAD phenotyping and prognosis

BOS is characterized by a progressive obstructive airflow limitation with obstructive spirometry and without persistent radiological opacities or total lung capacity decline. RAS is defined as a decline in spirometry with a restrictive pattern and total lung capacity decline ≥10% compared with baseline, along with persistent opacities on chest imaging. In the mixed phenotype, an obstructive-restrictive spirometry pattern is observed with persistent opacities.^3^ Computerized tomography features in BOS include air trapping and mosaicism, bronchial wall thickening, and bronchiectasis.^96^ If opacities are seen, this should prompt investigation for infection and rejection. Pleuro-parenchymal abnormalities on computerized tomography are diagnostic of RAS. Ground glass opacities are seen mostly in the earlier stages of RAS and consolidation in the later stages. Once diagnosed, BOS carries a median survival of approximately 3 to 5 years, compared to a median survival of 6 to 18 months in RAS.^97^ The “mixed” phenotype describes patients who have a combination of obstructive and restrictive ventilatory defects and persistent opacities. It also includes patients who may have transitioned from BOS to RAS. The final category (“undefined”) encompasses patients who do not clearly fit another phenotype. RAS is described in around 30% of CLAD patients and carries a poorer prognosis than BOS.^97^ Infection post-transplant, gastroesophageal reflux disease, and early episodes of ACR as described above are recognized risk factors for CLAD.^98^ It is important to consider other causes of changes in spirometry, including infections and rejection, anastomotic strictures/malacia, and disease recurrence, in addition to nonallograft-related causes, including obesity, ascites, and pleural or diaphragm disease.

### Biomarkers in CLAD

CLAD remains a major obstacle impairing outcomes in lung transplant. Alongside better characterization of CLAD, there is a growing interest in identifying potential biomarkers that might assist in the earlier detection of CLAD and characterization of CLAD phenotypes.^99^ There is evidence to suggest that serum and BAL eosinophilia^100^, ^101^ can be associated with poorer outcomes post lung transplant. An increase in NK cells (natural killer cell group 2 isoform C receptor) in BAL, which occurs in response to infection with CMV, is associated with an increase in the risk of developing CLAD and mortality.^102^ IL-6 has been shown to be elevated in BAL of patients with CLAD^103^ and elevated in patients with RAS compared with controls and patients with BOS.^104^ There is a need for more rigorous research to explore the potential role of biomarkers.

### Treatment of CLAD

The treatment options for CLAD are limited; conceptually, the approach can be considered a form of “host versus graft” disease, and attempts to address the immune response are in part based on the protocols used in pulmonary graft vs host disease.^105^ In established CLAD, the initial treatment involves an initial course of increased steroids, often high-dose intravenous methylprednisolone. If patients are not already on azithromycin prophylaxis, a prolonged course (at least 8 weeks) should be completed. Azithromycin-responsive allograft dysfunction is seen in about 10% to 15% of cases,^83^ and there is evidence to show increased CLAD-free survival with the use of azithromycin.^74^, ^94^

Further treatment options target the adaptive immune response. These include lymphoid depletion via total lymphoid irradiation, antithymococyte globulin or alemtuzumab, increasing the Treg population with extracorporeal photopheresis. Antifibrotic agents, including nintedanib and pirfenidone, have also been investigated,^106^ pirfenidone had no apparent impact in a single-center study in patients with CLAD.^107^ In rare cases, retransplantation may be offered.^105^ Retransplant for RAS has a higher mortality than retransplant for BOS.^108^ A retrospective analysis of the United Network for Organ Sharing Database of patients aged 18 or over undergoing a second double lung transplant found significantly worse 5-year survival when compared to the primary transplant cohort.^109^ In a pediatric cohort, retransplantation in the first year was associated with poorer outcomes when comparing graft survival with a 5-year survival of 28% for retransplant patients vs 44% for primary transplant recipients.^110^

Montelukast is associated with attenuation of the rate of FEV1 decline in recipients with late-onset BOS stage 1.^111^ Another single-center study demonstrated a significant attenuation in the rate of FEV1 decline in a substantial proportion of patients with established CLAD.^112^ Extracorporeal photopheresis appears to have potential as an effective second-line treatment in CLAD patients; in one study extracorporeal photopheresis responders demonstrated improved progression-free survival^113^ and clinical trials are ongoing.^114^ Alemtuzumab may be useful in refractory ACR but its use appears limited in CLAD.^115^ Other treatments being considered include newer immunomodulatory molecules such as ruxolitinib and baricitinib (JAK1/2 inhibitors), belumosudil (ROCK2 inhibitor), tyrosine kinase inhibitors, and monoclonal antibodies specific to cytokines and receptors in the T-cell cascade.^105^ Outcome data for these are currently limited with a lack of organ-specific responses in the trials published to date.^105^

## Future focus

The focus for research into CLAD should be toward prevention. Once memory T-cell populations exist, it is extremely difficult to remove them from the immune system in a targeted fashion, and current options to modify the actions of these cells (through depletion or increasing regulatory populations) are imprecise. As an epithelial surface facing an external environment, the immune system in the lung will inevitably be primed, and the routine challenges of the lung environment (viral infections, inhaled pollutants) result in cell lysis and increased exposure to non-self antigens. Interrupting the initial steps in T-cell recognition of foreign epitopes, either at the formation of the T-cell receptor or in the initial intracellular cascades that result in subsequent cell signaling, in theory, should provide the ideal target for intervention. Pediatric lung transplant physicians should follow the examples of our colleagues in oncology to prospectively enroll all children into trials of agents (such as JAK inhibitors or anti-IL17 monoclonals) that may prevent CLAD. Although the number of pediatric transplants per year is relatively few, the high incidence of CLAD means that trials may not need excessive numbers of participants to be recruited; this approach has been previously demonstrated in a randomized controlled trial of induction therapy in pediatric lung transplant recipients^60^ and adult physicians' approach to treatment of CLAD^114^ serves as an exemplar. This will require international collaborations and modern approaches, including registry-based trials.

## CRediT authorship contribution statement

All authors contributed to the manuscript. A.S. and R.B. were responsible for the conceptual design of the manuscript. A.S. and R.B. conducted a literature search and drafted the manuscript. All authors edited and reviewed the manuscript. All authors approved the final manuscript.

## Funding

Not applicable.

## Declaration of Competing Interest

The authors declare that they have no known competing financial interests or personal relationships that could have appeared to influence the work reported in this paper.
